# Transforming Diabetes Care: The Expanding Role of DPP-4 Inhibitors in Cardiovascular and Renal Protection

**DOI:** 10.3390/medicina60111793

**Published:** 2024-11-01

**Authors:** Francisco Epelde

**Affiliations:** Medicine Department, Institut d’Investigació i Innovació Parc Taulí I3PT, Parc Taulí Hospital Universitari, 08208 Sabadell, Spain; fepelde@gmail.com

## 1. Introduction

The approach to managing type 2 diabetes mellitus (T2DM) has significantly progressed, evolving beyond strict glycemic control to addressing the wider complications associated with the disease, including cardiovascular disease (CVD) and chronic kidney disease (CKD) [1,2]. These conditions are prevalent in diabetic patients, contributing to high rates of morbidity and mortality. Dipeptidyl peptidase-4 (DPP-4) inhibitors, known primarily for their glucose-lowering effects, have become integral in the therapeutic regimen for T2DM due to their low risk of hypoglycemia and weight neutrality, making them particularly suitable for a diverse patient population [3,4]. In recent years, research has uncovered potential cardiovascular and renal protective effects, broadening the scope of their therapeutic application [5,6,7]. Given the critical need to improve outcomes for high-risk populations, this Special Issue encourages the submission of original research, reviews, and clinical studies focused on the expanding therapeutic potential of DPP-4 inhibitors.

## 2. DPP-4 Inhibitors: Shifting Beyond Glycemic Control

DPP-4 inhibitors control glucose effectively by preventing the breakdown of incretin hormones, thus enhancing insulin secretion and inhibiting glucagon release [8,9]. Unlike older antidiabetic medications, DPP-4 inhibitors achieve this without causing weight gain or significant hypoglycemia, making them particularly beneficial for patients with cardiovascular and renal comorbidities [10]. Recent research highlights that DPP-4 inhibitors’ impact extends beyond glycemic control, with studies indicating potential benefits for cardiovascular and renal health, particularly in patients with heart failure and CKD [11,12]. This multifaceted therapeutic role positions DPP-4 inhibitors as valuable agents in managing T2DM and its associated complications. We invite researchers to explore this area further to uncover the non-glycemic advantages that may enhance patient outcomes.

## 3. DPP-4 Inhibitors and Heart Failure: A Critical Examination

Patients with diabetes are at an increased risk of developing heart failure (HF), a condition that complicates diabetes management and worsens prognosis [13]. The cardiovascular safety of antidiabetic drugs is thus an area of considerable interest, especially following the SAVOR-TIMI 53 trial, which reported an unexpected increase in HF hospitalizations among patients treated with saxagliptin [14]. However, subsequent trials, including EXAMINE, which examined alogliptin, and TECOS, which studied sitagliptin, did not observe a similar increase in HF risk, suggesting that the cardiovascular effects of DPP-4 inhibitors may vary by agent and patient profile [15,16,17,18]. These diverse findings underscore the need for additional research to clarify the cardiovascular effects of DPP-4 inhibitors and identify patient subsets that may benefit or experience increased risk. Exploring these complex relationships further will help inform the safe integration of DPP-4 inhibitors in HF management for diabetic patients, providing clinicians with greater insights into patient-tailored therapeutic strategies.

## 4. Renal Protection: A Promising, Yet Incomplete, Picture

CKD remains a challenging and often devastating complication of diabetes, with limited treatment options as the disease progresses [19]. Certain DPP-4 inhibitors, such as linagliptin, can be administered in patients with advanced CKD without dose adjustments, making them a favorable choice for diabetic patients with renal impairment [20]. While DPP-4 inhibitors have demonstrated efficacy in slowing the progression of albuminuria, their impact on renal outcomes remains modest, particularly when compared to the more potent renoprotective effects of sodium-glucose co-transporter 2 (SGLT-2) inhibitors [21,22]. Nonetheless, the anti-inflammatory and anti-fibrotic properties of DPP-4 inhibitors suggest potential long-term benefits, a hypothesis that warrants further investigation [23]. The CARMELINA trial, which evaluated linagliptin, confirmed its renal safety but highlighted the need for additional studies to assess its potential for direct renal protection [24]. We encourage research submissions on DPP-4 inhibitors in combination with other renal protective agents, such as SGLT-2 inhibitors or GLP-1 receptor agonists, to further optimize CKD management in diabetic patients.

## 5. DPP-4 Inhibitors in Cardiovascular-Renal Strategy: A Multifactorial Approach

As diabetes management continues to evolve, DPP-4 inhibitors should be considered within a broader cardiovascular and renal strategy [25]. For diabetic patients at low cardiovascular or renal risk, DPP-4 inhibitors remain an excellent choice for glycemic control due to their favorable safety profile, convenience, and efficacy across diverse patient populations, including the elderly [26,27]. In higher-risk patients with established heart failure or advanced CKD, treatment decisions become more nuanced, and DPP-4 inhibitors may be best used in combination with other agents, such as SGLT-2 inhibitors, which provide greater cardiovascular and renal benefits [28,29]. The future of diabetes management likely lies in such combination therapies, with DPP-4 inhibitors being integrated into multi-drug regimens to achieve optimal outcomes for patients facing complex clinical challenges. Studies investigating these combined therapeutic strategies and their impact on cardiovascular and renal health will be of particular interest.

## 6. A Call for Research and Contributions

This Special Issue seeks to expand the current knowledge on DPP-4 inhibitors in diabetes care. Researchers are invited to submit articles that delve into the cardiovascular and renal effects of DPP-4 inhibitors, emphasizing studies that provide mechanistic insights, real-world applications, and innovative combination therapies. Through these contributions, we aim to advance our understanding of DPP-4 inhibitors and improve patient care by addressing the dual challenges of cardiovascular and renal disease in diabetes management.
